# Gastric foreign body: a comb

**DOI:** 10.1002/ccr3.957

**Published:** 2017-04-17

**Authors:** Wen‐Jung Chang, Wen‐Yi Chiu

**Affiliations:** ^1^ Division of General SurgeryDepartment of SurgeryKaohsiung Armed Forces General HospitalKaohsiungTaiwan; ^2^ Departments of Family MedicineKaohsiung Armed Forces General HospitalKaohsiungTaiwan

**Keywords:** Comb, computed tomography, exploratory laparotomy

## Abstract

The ingestion of a foreign body is a common occurrence. Psychiatric patients and prisoners may swallow objects on purpose. Occasionally emergent exploratory laparotomy may be indicated if the diagnosis is uncertain, and emergent removal of the foreign body may be needed to prevent substantial risk of serious complications including perforation, fistula, or gastrointestinal bleeding.

Quiz question: What is the specific finding from the image study?

A 38‐year‐old woman with major depressive disorder was admitted to our psychiatric ward with sudden onset of chest tightness, shortness of breath, and epigastric pain with nausea and vomiting. No abnormal findings were noted on physical examination, and after the interview, she claimed that she had swallowed a comb for self‐mutilation. Radiographs of the abdomen and the chest were normal. Coronal reconstructed noncontrast abdominal computed tomography (CT) performed because of persistent discomfort revealed a comb‐shaped opacity within the stomach (Fig. 1). The image was consistent with a subsequent endoscopy finding of a comb stuck in the esophagogastric junction (Fig. 2). Following an exploratory laparotomy, the comb, measuring 18 × 2.4 × 0.4 cm, was removed by gastrostomy. Postoperative recovery was uneventful.

**Figure 1:**
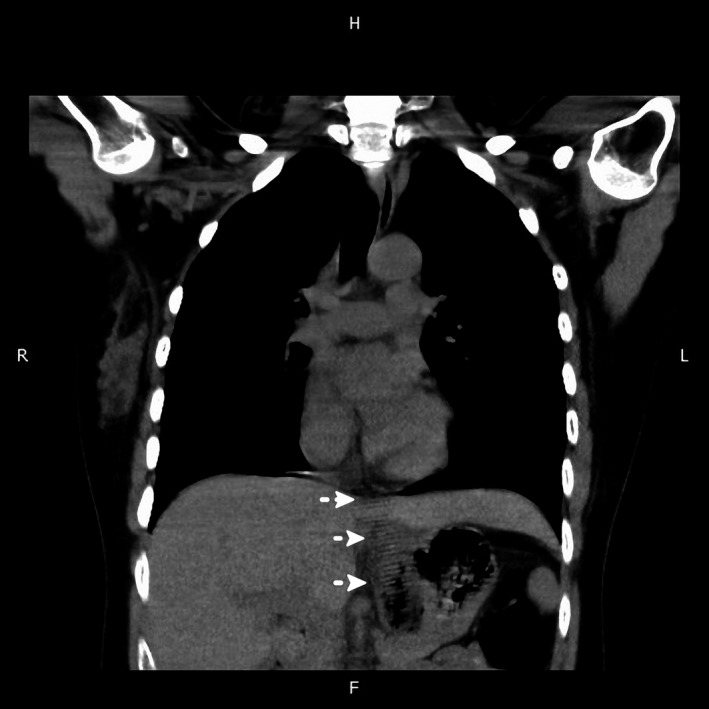
Coronal reconstructed noncontrast abdominal computed tomography revealing a comb‐shaped opacity within the stomach (white arrow).

**Figure 2:**
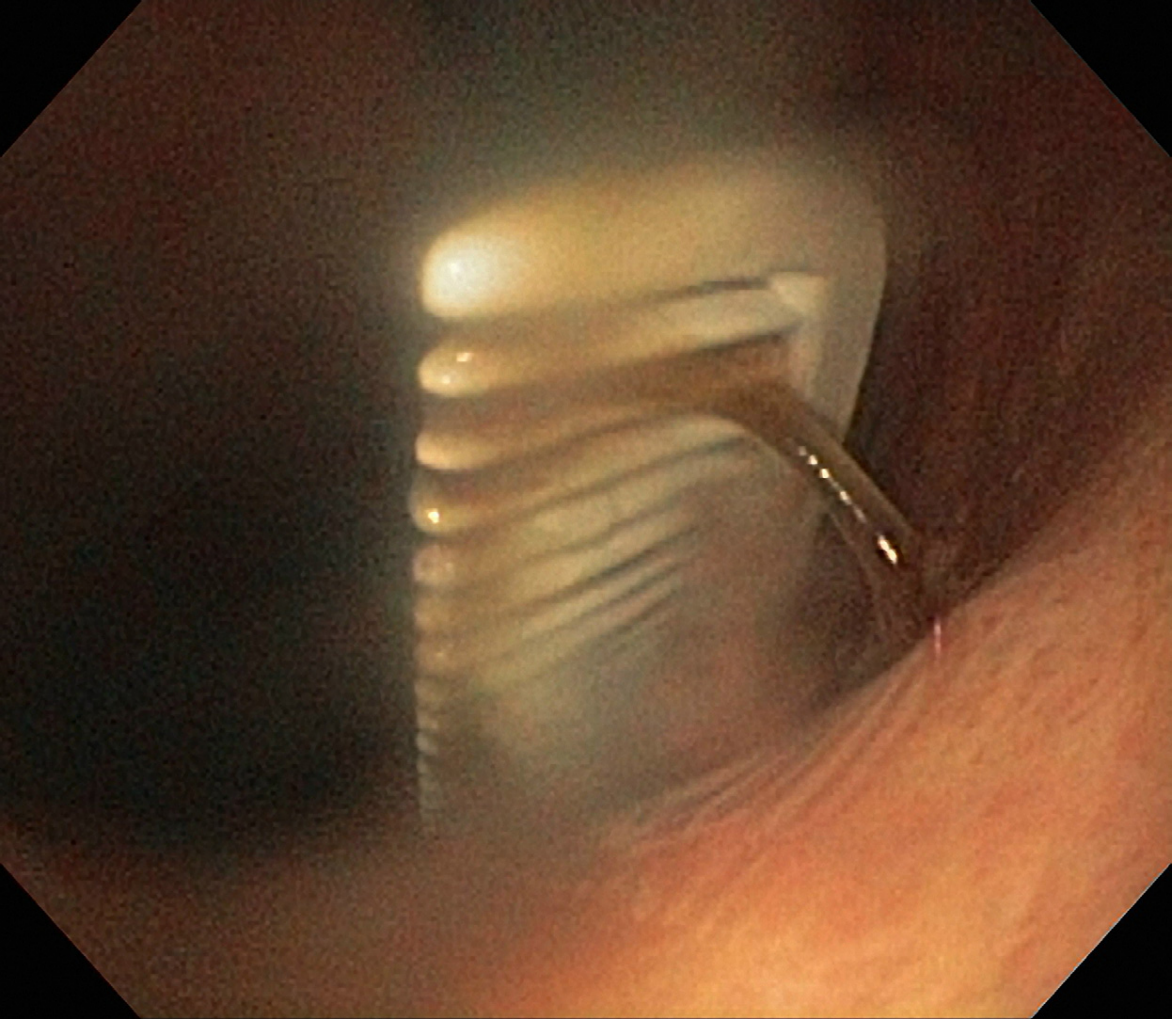
Esophagogastroduodenoscopy revealing a comb stuck in the esophagogastric junction.

The ingestion of foreign bodies is a common phenomenon. Up to 80% of patients are children, elderly patients with cerebrovascular diseases, mentally retarded, or alcohol dependents. Psychiatric patients and prisoners may swallow objects on purpose 1.

Approximately 80–90% of ingested foreign bodies pass through the gastrointestinal tract spontaneously without complications. Endoscopic intervention may be required in 10–20% of patients, and <1% of cases need surgical treatment 1, 2. In this patient, emergent exploratory laparotomy with gastrostomy was performed for removal of the comb to prevent serious complications including perforation, fistula, or gastrointestinal bleeding. Because foreign bodies may mimic artificial defects in plain imaging or CT, prompt endoscopy is indicated in patients suspected of ingesting foreign objects.

## Conflict of Interest

The authors report no conflicts of interest relevant to the manuscript.

## Authorship

WJC: involved in literature search, concept, and drafting. WYC: involved in literature search, concept, and revision of article. The authors report no disclosures relevant to the manuscript.
